# *Rugitermesursulae* (Isoptera, Kalotermitidae), a new drywood termite from the Caribbean coast of Colombia

**DOI:** 10.3897/zookeys.1057.65877

**Published:** 2021-08-25

**Authors:** Robin Casalla, Rudolf H. Scheffrahn, Judith Korb

**Affiliations:** 1 Universidad del Norte, Departamento de Química y Biología. Kilómetro 5 Antigua vía Puerto Colombia, Barranquilla Colombia Universidad del Norte Barranquilla Colombia; 2 Fort Lauderdale Research and Education Center, University of Florida, 3205 College Avenue Davie, Florida 33314, USA University of Florida Davie United States of America; 3 Universität Freiburg, Evolutionary Biology & Ecology. Hauptstrasse 1, Freiburg 79104, Germany Universität Freiburg Freiburg Germany

**Keywords:** DNA barcoding, imago, northern Colombian coast, soldier, taxonomy, tropical dry forest

## Abstract

*Rugitermesursulae***sp. nov.** is described from a sample collected inside a dead branch in a tropical dry forest of Colombia’s Caribbean coast using molecular information and external morphological characters of the imago and soldier castes. *Rugitermesursulae***sp. nov.** soldiers and imagoes are the smallest among all described *Rugitermes* species. The imago’s head capsule coloration is dark castaneous, while the pronotum is contrastingly pale yellow. Our description includes soldier characters, such as subflangular elevation and shape of the antennal sockets, that can help in identification of samples lacking imagoes.

## Introduction

**Figure 1.**
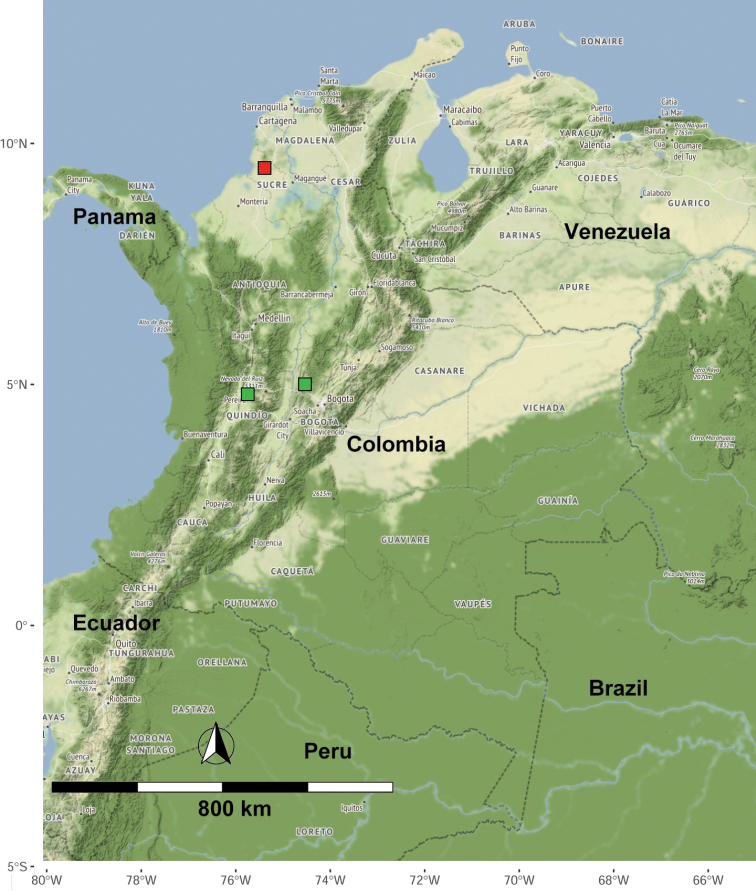
Type localities of *Rugitermes* species described from Colombia. *Rugitermesursulae* sp. nov. (red square) and *R.tinto* (green squares).

Records of termites from Colombia have increased in recent years (Scheffrahn 2010; Casalla et al. 2016a, b; Postle and Scheffrahn 2016; Castro et al. 2018; Pinzón and Castro 2018; Casalla and Korb 2019; Castro and Scheffrahn 2019; Scheffrahn 2019; Pinzón et al. 2020). The “Los Primates” area in the mountains of the municipality of Colosó, Sucre, is one of the best-preserved areas of tropical dry forest on the Colombian Caribbean coast (Pizano and García 2014) (Fig. 1).

*Rugitermes* Holmgren, 1911, is a genus from the family Kalotermitidae, mainly found in the Neotropics (Krishna et al. 2013; Scheffrahn 2015; Scheffrahn and Carrijo 2020). Twelve *Rugitermes* species are currently known from South America, including the most recently described *Rugitermestinto* Scheffrahn & Pinzón, 2020, *Rugitermesaridus* Scheffrahn, 2020, *Rugitermesrufus* Scheffrahn, 2020 and *Rugitermesvolcanensis* Scheffrahn, 2020 (Scheffrahn and Carrijo 2020; Scheffrahn and Pinzón 2020) from the Andean region (Table 1).

**Table 1.** Comparison of head measurements (mm) and coloration of soldiers and imagoes of different *Rugitermes* species.

No.	Species	Distribution*	Soldier		Imago				Reference
			Head width	Mean	Head width	Mean	Head color	Pronotum color	
1	*R.ursulae* sp. nov.	SA (Colombia)	1.12–1.22	1.17	1.10–1.20	1.15	Dark castaneous	Pale yellow	This publication
2	* R. flavicinctus *	SA (Guyana)	1.19–1.35	1.27	1.19	1.19	Black	Yellow	Emerson 1925
3	* R. rufus *	SA (Bolivia)	1.16–1.56	1.42	1.19–1.26	1.23	Reddish		Scheffrahn and Carrijo 2020
4	* R. magninotus *	SA (Guyana, Peru)	1.38–1.48	1.43	1.25	1.25	Black	Yellow	Emerson 1925
5	* R. volcanensis *	SA (Bolivia)	1.24–1.88	1.54	1.21	1.21	Black		Scheffrahn and Carrijo 2020
6	* R. athertoni *	OC (Polynesia)	1.47–1.73	1.60	1.26	1.26	Brown/Black		Light 1932
7	* R. aridus *	SA (Peru)	1.44–1.72	1.60	NA	NA	Black	Yellow	Scheffrahn and Carrijo 2020
8	* R. niger *	SA (Brazil)	1.50–1.80	1.65	1.26–1.34	1.30	Black		Oliveira 1979
9	* R. kirbyi *	CA (Costa Rica, Panama)	1.65	1.65	1.60	1.60	Black	Yellow	Snyder 1926
10	* R. tinto *	SA (Colombia)	1.52–1.90	1.72	1.31	1.31	Black	Yellow	Scheffrahn and Pinzón 2020
11	* R. unicolor *	CA (Honduras)	1.80	1.80	1.60	1.60	Yellow-brown	Yellow	Snyder 1952
12	* R. panamae *	CA (Panamá)	1.8–2.0	1.90	1.4	1.40	Black	Yellow	Snyder 1925
13	* R. bicolor *	SA (Guyana)	1.80–2.07	1.94	1.51	1.51	Black	Yellow	Emerson 1925
14	* R. occidentalis *	SA (Argentina)	2.00	2.00	1.35	1.35	Black	Brown	Silvestri 1901
15	* R. laticollis *	SA (Bolivia, Ecuador)	1.83–2.43	2.06	1.5–2.00	1.68	Black		Snyder 1957; Scheffrahn 2015
16	* R. costaricensis *	CA (Costa Rica)	2.10	2.10	1.80	1.80	Yellow-brown	Yellow	Snyder 1926
17	* R. nodulosus *	SA (Brazil)	NA	NA	NA	NA	Black	Yellow	Hagen 1858
18	* R. rugosus *	SA (Brazil)	1.21 (1.7?)	NA	NA	NA	Black		Hagen 1858

NA = Not available * CA = Central America, OC = Oceania, SA = South America Soldier’s scale
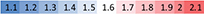
Imago’s scale
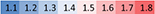

*Rugitermes* species have few species-specific diagnostic characters. The dorsal antennal ridge and the anterolateral corner of the frontal ridge of the soldier head, the size of the eyes of imagoes and soldiers as well as the imago’s head shape can provide useful information to describe a new species (Krishna et al. 1961; Scheffrahn and Carrijo 2020).

Here, we describe the soldiers and imagoes of *Rugitermesursulae* sp. nov. from a sample collected inside a dead branch in the tropical dry forest of Colombia’s Caribbean coast. In addition, we performed molecular analyses based on the marker COII (cytochrome oxidase II) and including representatives of other genera of Kalotermitidae to support species description.

## Materials and methods

### Study sites and sampling

In July 2014, a survey was done in the tropical dry forest of Colombia’s Caribbean coast (Casalla and Korb 2019). One sample of a new *Rugitermes* species was collected, which was preserved in 100% ethanol for molecular DNA analysis and in 80% ethanol for museum curation.

### Identification and genetic analysis

*Rugitermesursulae* sp. nov. was compared with *Rugitermes* samples from the University of Florida Termite Collection (UFTC), USA, and with descriptions and measurements from the literature (Hagen 1858; Silvestri 1901; Emerson 1925; Snyder 1925, 1926, 1952, 1957; Light 1932; Oliveira 1979; Scheffrahn 2015, 2020; Scheffrahn and Pinzón 2020). Specimens of *R.ursulae* were sequenced for a fragment of the molecular marker COII for genetic comparisons. Total DNA was extracted from the heads of pseudergates (‘false workers’) using the CTAB protocol (Fuchs et al. 2003). Due to limited availability of mitochondrial gene sequence data for species of Kalotermitidae at the National Center for Biotechnology Information (NCBI), we restricted our sequencing to the COII fragment (~740 bp), for which most data were available. We also sequenced a *Rugitermes* specimen from Colombia (*Rugitermes* ADD 2015-29), which lacks imagoes. PCRs and sequencing were performed following the protocol in Hausberger et al. (2011).

We considered COII sequences for 26 species of Kalotermitidae (if available three species per genus) and the woodroach, *Cryptocercuspunctulatus*, as an outgroup (Suppl. material 1: Table S1). Thus, we covered 18 of the 22 known genera of Kalotermitidae worldwide. Sequences were aligned at the nucleotide level with the MUSCLE alignment algorithm as implemented in MEGA X v.10.1.8 with default settings (Kumar et al. 2018).

We inferred a phylogenetic tree based on the maximum likelihood (ML) approach. We selected the best fitting model using ModelFinder (Kalyaanamoorthy et al. 2017), which includes the free rate variation as implemented in IQ-TREE v. 1.6.12 (Nguyen et al. 2015). The selected model and parameter setting was TIM2+F+I+G4 according to the corrected Akaike Information Criterion (AICc, Kalyaanamoorthy et al. 2017). We performed 20 independent ML tree searches, 10 with a random starting tree and 10 with a parsimony starting tree, using the selected model, random seeds, and otherwise defaults settings. We compared the tree topologies among all inferred ML trees using Unique tree v. 1.9 (kindly provided by T. Wong and available upon request, Australian National University) as described in Misof et al. (2014), which resulted in one unique topology among the 20 inferred trees. We selected the ML tree with the best log likelihood value (including branch length). For statistical support, we performed 1000 non-parametric, slow bootstrap (BS) replicates with random starting trees and mapped statistical support onto the best ML tree using IQ-TREE. We ensured bootstrap convergence applying ‘posteriori bootstrap criteria’ (see Pattengale et al. 2010) using majority rule (MR) and 10,000 pseudo-replicates. Convergence was checked in five runs independently with different random seeds, all bootstrap convergence checks were performed with default settings in RaxML v.8.2.11 (Stamatakis 2014). Bootstrap convergence was achieved in each run after 5000 BS replicates. The best ML with statistical BS support tree was visualized using Seaview v.5.0.4 (Gouy et al. 2010).

We calculated *p*-distances between COII sequences with MEGA X v.10.1.8 (Kumar et al. 2018) using the following parameters: *p*-distance model, variance estimation model with 10,000 bootstrap replicates; the rate variation among sites (ASRV) was modeled with a gamma distribution (+G).

### Imaging and measurements

Specimens were suspended in hand sanitizer and images were taken with a Leica M205 C stereomicroscope coupled to a Leica MC190 HD digital camera. Helicon Focus software was used to stack pictures. Measurements were done following Roonwal (1969).

## Results

### Taxonomy

#### Rugitermes ursulae

Taxon classificationAnimaliaBlattodeaKalotermitidae

Casalla, Scheffrahn & Korb sp. nov.

AF1BB92C-33B0-5713-9381-55CDDF70B30F

http://zoobank.org/F5008BCF-2569-4654-81ED-B18815044CF9

**Figure 2.**
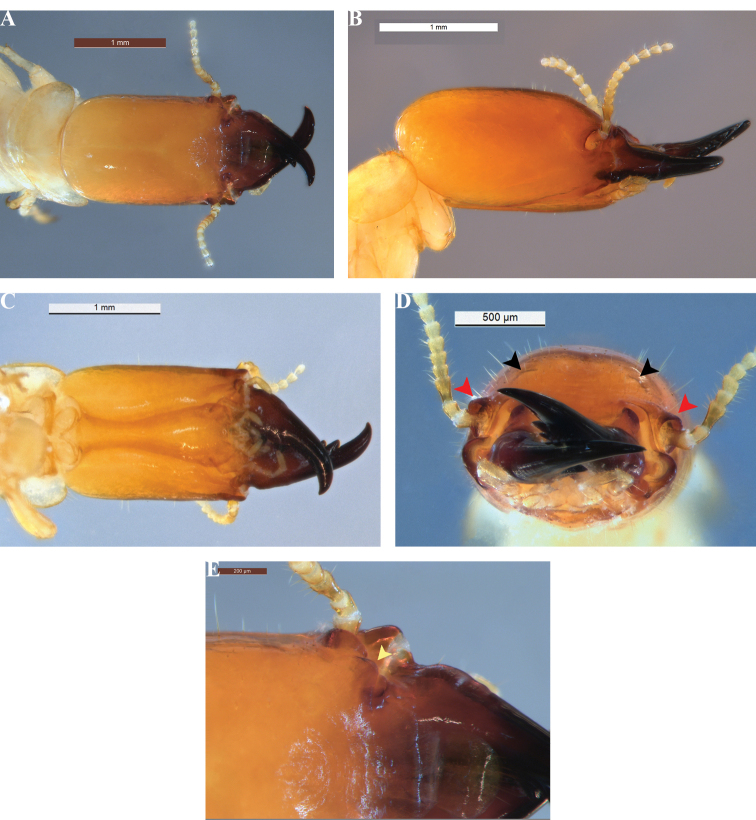
*Rugitermesursulae* sp. nov. soldier (holotype) head and pronotum **A** dorsal **B** lateral **C** ventral view **D** frontal view (black arrows mark inconspicuous subflangular elevation, red arrows mark margins of antennal carinae) **E** anterior dorsal (yellow arrow marks frontolateral ridge).

**Figure 3.**
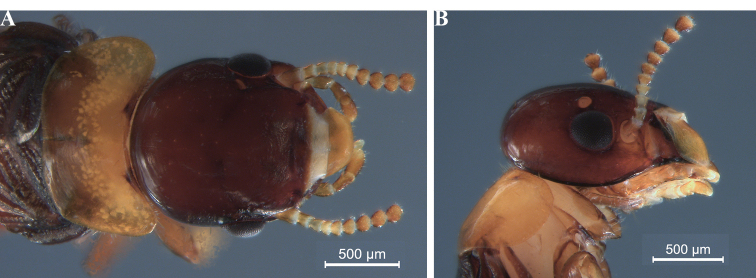
*Rugitermesursulae* sp. nov. imago head and pronotum **A** dorsal **B** lateral view.

Figs 2 , 3 ; Tables 1 , 2 , 3

##### Material examined.

***Holotype*** soldier. Colombia: Colosó, Sucre (9.5435, -75.34884), 400 meters a.s.l., 11.JUL.2014, R. Casalla, ADD-2014-10A. ***Paratypes.*** One additional soldier, 12 pseudergates, a pair of reproductive, same colony sample as holotype ADD-2014-10B. Voucher specimen are held at the Universidad del Norte, Colombia. Holotype soldier (ADD-2014-10A) and one reproductive paratype of *Rugitermesursulae* sp. nov. (ADD-2014-10B-1) will be deposited at the Natural History Museum of the Alexander von Humboldt Institute of Bogotá (MIAvH, Colombia) and a paratype soldier (ADD-2014-10B-2) and reproductive (ADD-2014-10B-3) at the collection of the American Museum of Natural History, New York. (AMNH, USA). Pseudergates (‘false workers’) of *R.ursulae* will be part of the collection of termites of the Department of Chemistry and Biology at the University del Norte.

##### Diagnosis.

The soldier of *R.ursulae* sp. nov. is the smallest of all congeneric soldiers (Fig. 2). The size of the head is remarkably small (Tables 1, 2). The pronotum width is almost twice its length, for both imago and soldier. Antennal sockets are pronounced, protruded, and rectangular in the soldier. The soldier of *R.ursulae* sp. nov. can be distinguished by its small subflangular elevation.

**Table 2.** Measurements (mm) of *Rugitermesursulae* sp. nov. soldiers (*N* = 2).

Character	Holotype	Measurements
Head length with mandibles	2.81	2.81, 2.90
Head length to lateral base of mandibles	1.87	1.87, 1.99
Head width max.	1.12	1.12, 1.22
Head height with postmentum	1.06	1.06, 1.20
Postmentum width min.	0.22	0.22, 0.24
Postmentum length	1.34	1.34, 1.38
Pronotum width	1.27	1.27, 1.39
Pronotum length	0.63	0.63, 0.70
Third antennal article length	0.16	0.16, 0.17
Left mandible length (from dorsal condyle)	1.17	1.17, 1.22
Frontal angle (° degrees)	34	29, 34

The imago of *R.ursulae* sp. nov. is also the smallest of all *Rugitermes* species (Fig. 3; Tables 1, 3). The imago of *R.ursulae* sp. nov. has disproportionally large eyes and ocelli in relation to head dimensions, when compared with another small *Rugitermes* species, *R.flavicinctus*, which is known from Guyana (Table 1; Fig. 3). *Rugitermesursulae* sp. nov. is the only *Rugitermes* imago with a dark-castaneous head capsule and pale-yellow coloration of the pronotum.

**Table 3.** Measurements (mm) of *Rugitermesursulae* sp. nov. dealated imagoes (*N* = 2).

Character	Measurements
Eye diameter max.	0.30, 032
Ocellus diameter max.	0.09, 0.10
Head width (max. with eyes)	1.10, 1.20
Head length to tip	1.44, 1.49
Head height	0.78, 0.87
Pronotum width max.	1.25, 1.27
Pronotum length min.	0.64, 0.70

##### Type locality.

“Los primates” Colosó, Sucre, Colombia (Fig. 1, Suppl. material 2: Fig. S1)

##### Description.

***Soldier*** (Fig. 2, Table 2). Head capsule, in dorsal view, light yellowish orange. Occiput and posterior vertex grading from pale orange to yellow orange towards frons. Postmentum concolorous with head capsule; narrowest at posterior third (Fig. 2C). Pronotum pale yellow, nearly transparent toward lateral margins. Mandibles dark reddish brown near base, grading to black from mid-length to tips. Third antennal article clavate, more pigmented than other articles; article formula 2<3>4=5. Pronotum twice as wide as long, anterior margin shallowly concave, anterolateral corners evenly rounded with a few bristles and scattered setae in posterolateral margins. Postmentum concolorous with head capsule; narrowest at posterior third (Fig. 2C). Eye spots barely discernable, forming pale blotches behind antennal carinae. In dorsal view, head capsule rectangular with lateral margins parallel. Frons angled ca 30° below plane of vertex (Fig. 2B); frons weakly concave with undulating rugosity in middle extending to postclypeus (Fig. 2E). In frontal view, shallow elevations on each side of frontal margin; about a dozen medium to long setae above and lateral to each elevation (Fig. 2D). Frontolateral ridges about 85° in dorsal view; corners evenly rounded (Fig. 2E). Antennal carinae with projecting dorsal and posterior margins not exceeding head width; anterior margins below frontolateral ridges. Mandibles with weak basal humps; outer margins of blades straight from humps to distal fourth. First marginal tooth of left mandible three-fourths from tip; directed forward. First marginal tooth of right mandible at basal third.

***Imago*** (Fig. 3, Table 3). Head capsule dark castaneous; pronotum pale yellowish, contrasting sharply with head capsule. Eyes small, ellipsoid. Ocellus hyaline, nearly circular, separated from eye by one-third its diameter. Antennae articles 2–4 pale yellow; first darker, fifth and beyond progressively darker. Antennae with at least 10 articles (broken), formula 1>2=3>4. Head vertex and frons with few short setae. Pronotum twice as wide as long. Pronotum wider than head capsule; anterior margin straight, posterolateral corners evenly rounded with scattered bristles, posterior margin narrowly concave. Legs light brown grading to pale yellow toward tibia. Arolium present.

##### Distribution and biological observations.

‘Los Primates’ is located in the mountains of the municipality of Colosó, Sucre. It is a regional forest reserve created in 1983, containing primary and secondary tropical dry forest. The mean annual temperature is 26.7 °C (min: 25.8 °C; max: 27.8 °C) with an annual precipitation of around 1337 mm (INDERENA, 1983; Hijmans et al. 2005). The specimens of *Rugitermesursulae* sp. nov. were collected from a small, dry branch (ca 12 mm in diameter) of a leafless bush (Suppl. material 2: Fig. S1).

##### Etymology.

“Ursulae” derived from a diminutive of the Latin *ursa*, which means “little bear”, in line with the small size of the species. Ursula is also the name of José Arcadio Buendía’s wife in the novel “One hundred years of solitude” written by Gabriel García Márquez and represents an apology/symbolism for the spiritual engine, entrepreneurship, and hard and silent work of many women around the world.

##### Molecular analysis of the COII fragment.

**Figure 4.**
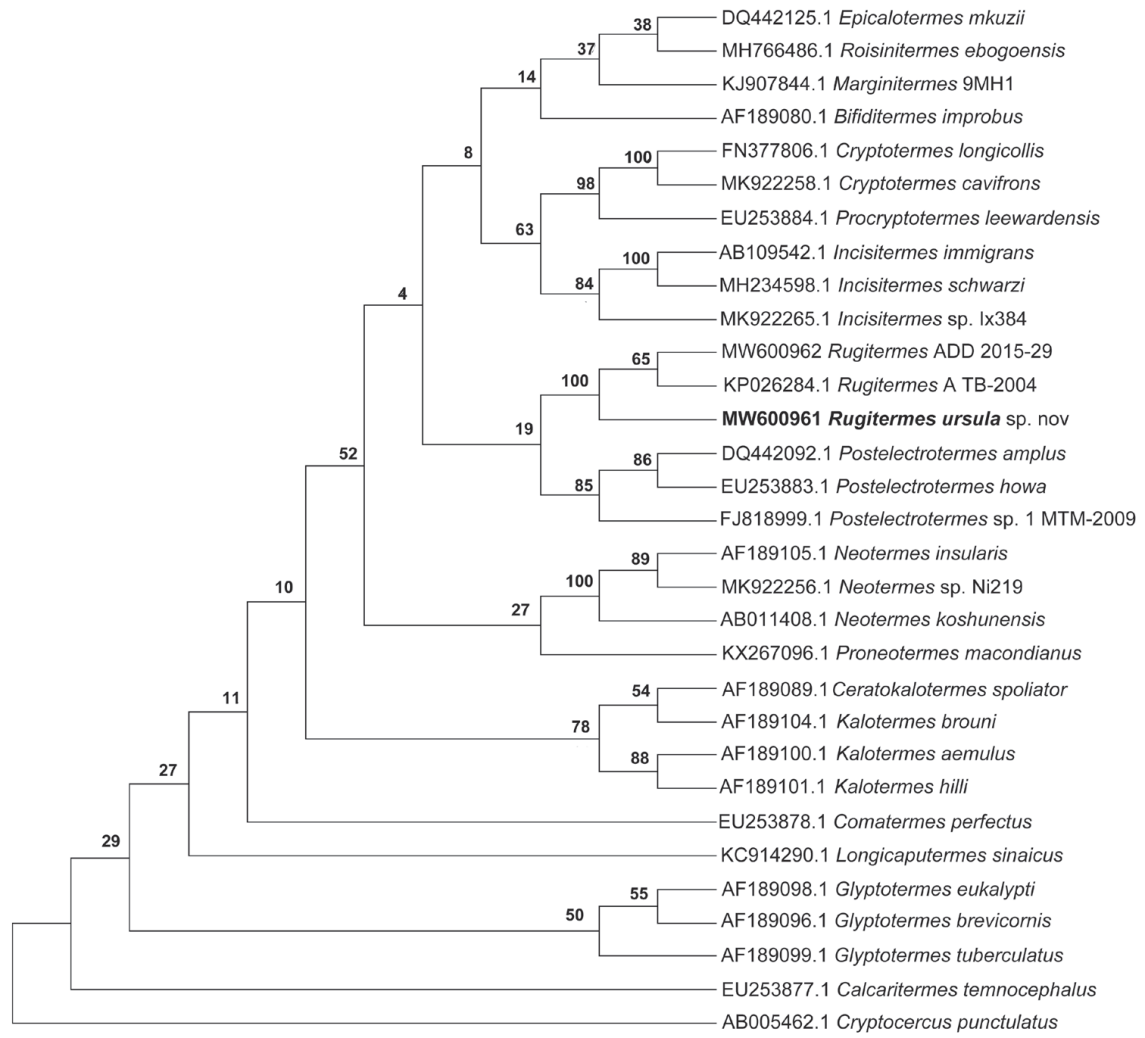
Maximum likelihood (ML) tree inferred from COII mtDNA gene sequences of 30 species of Kalotermitidae, including the woodroach, *Cryptocercuspunctulatus*, as outgroup. Numbers before the splits show statistical bootstrap support (BS). Terminals are labeled with the respective NCBI access numbers and genus or species name. *Rugitermesursulae* sp. nov. is shown in bold.

The topology and splits inferred from the multiple sequence alignment of the COII fragment for all Kalotermitidae genera available in NCBI, and including our new species, revealed a COIIML gene tree that clearly separated *R.ursulae* sp. nov. from the two other *Rugitermes* species with maximal BS support. Furthermore, it suggests that the genus *Rugitermes* is monophyletic (maximal BS support) and that it is the sister taxon of *Postelectrotermes*, however support values for the latter are low (19% BS; Fig. 4).

The *p*-distance analyses revealed that the barcode sequences most similar to *R.ursulae* (accession number: MW600961) belonged to *Rugitermes* sp. A TB-2014 (accession number: KP026284.1) and *Rugitermes* ADD 2015-29 (accession number: MW600962); they shared 87% and 86% sequence similarity (*p*-distance), respectively (Suppl. material 1: Table S2).

### Key to *Rugitermes* from Colombia

**Table:** 

1	Soldier with dark coloration of anterior head capsule. Anterolateral corners of frontal ridges project to form acute angles. Maximum soldier head width (mean) 1.72 mm; imago with a black head capsule; pronotum brownish orange, compound eye small, nearly circular. Ocellus very small; maximum head width (mean) 1.31 mm	*** Rugitermes tinto ***
–	Soldier with light yellowish orange of anterior head capsule. Anterolateral corners poorly developed. Maximum soldier head width (mean) 1.17 mm; imago with a dark-castaneous head capsule; pronotum pale yellowish, compound eye large, nearly circular. Ocellus small, oval shape; head width (mean) 1.15 mm	***Rugitermesursulae* sp. nov.**

## Discussion

The Caribbean Region of Colombia is rich in Kalotermitidae and the tropical dry forest supports a high species diversity for this family (Casalla et al. 2016a, b; Casalla and Korb 2019; Pinzón et al. 2020).

The phylogenetic relationships within the Kalotermitidae are not clearly resolved among the most recently discovered genera (Krishna 1961; Krishna et al. 2013). Molecular markers have helped to resolve the evolutionary relationships among termites (Bourguignon et al. 2015) but for some termite families such as the Kalotermitidae hurdles still persist. Thompson et al. (2000), using COII and cytochrome B sequences, inferred the phylogeny of Kalotermitidae of the Australian region. In addition to describing a new species, we used the generated COII sequences in an effort to obtain better resolution of the phylogenetic relationships among the Kalotermitidae. We use representatives of 18 genera from all over the world (Table 1). However, the relationships among genera could not be clearly resolved (Fig. 4). Clearly, more integrative studies that combine additional molecular, morphological, and ecological data are needed. For our study, phylogenetic reconstruction was mainly applied to test whether *R.ursulae* differed genetically from other *Rugitermes* species.

Molecular markers often allow a clear separation between *Rugitermes* congeners (Scheffrahn and Carrijo 2020). In line with this, *R.ursulae* sp. nov. was clearly separated from the other *Rugitermes* species in our study (Fig. 4). However, it is generally difficult to describe new *Rugitermes* species when only a few specimens are available. However, the anterolateral ridges of the frons seem to be good diagnostic markers in *Rugitermes* (Scheffrahn and Carrijo 2020). This differed clearly in *Rugitermesursulae* sp. nov. compared to described congeners. We identified the subflangular region (frontal head view) and the angle of the frontolateral ridge to be species-specific traits of soldiers. In addition, the coloration and the head of size of the imagoes, which are the smallest among all *Rugitermes* species (Table 1), support the description of a new species.

Our study shows the importance of further surveys at isolated sites in the tropics as they continue to reveal many new species. This is also essential for phylogenetic studies to infer the evolutionary history of the Kalotermitidae, and any taxonomic lineage in a broad way.

## Supplementary Material

XML Treatment for Rugitermes ursulae

## Supplementary materials

Supplementary material 1 Tables S1, S2 Data type Sequence accession identifier and Measures of genetic distance Explanation note **Table S1.** GenBank accession numbers for COII gene sequences of Kalotermitidae used in this study. **Table S2.** Nucleotide *p*-distances for COII sequences between *Rugitermesursulae* sp. nov. (bold) and other species belonging to Kalotermitidae. *Cryptocercuspunctulatus* used as outgroup. https://binary.pensoft.net/file/580887This dataset is made available under the Open Database License (http://opendatacommons.org/licenses/odbl/1.0/). The Open Database License (ODbL) is a license agreement intended to allow users to freely share, modify, and use this Dataset while maintaining this same freedom for others, provided that the original source and author(s) are credited.Robin Casalla, Rudolf H. Scheffrahn, Judith Korb

Supplementary material 2 Figure S1 Data type Multimedia: Photo Explanation note Biome for *Rugitermesursulae* sp. nov. (Tropical dry forest, Colosó, Colombia, July 2014). https://binary.pensoft.net/file/580888This dataset is made available under the Open Database License (http://opendatacommons.org/licenses/odbl/1.0/). The Open Database License (ODbL) is a license agreement intended to allow users to freely share, modify, and use this Dataset while maintaining this same freedom for others, provided that the original source and author(s) are credited.Robin Casalla, Rudolf H. Scheffrahn, Judith Korb

Supplementary material 3 Supplementary references Data type References https://binary.pensoft.net/file/580889This dataset is made available under the Open Database License (http://opendatacommons.org/licenses/odbl/1.0/). The Open Database License (ODbL) is a license agreement intended to allow users to freely share, modify, and use this Dataset while maintaining this same freedom for others, provided that the original source and author(s) are credited.Robin Casalla, Rudolf H. Scheffrahn, Judith Korb
