# Supplementary figures and images for: Rugitermesursulae (Isoptera, Kalotermitidae), a new drywood termite from the Caribbean coast of Colombia
Source: Zookeys. 2021 Aug 25;1057:23–36. doi: 10.3897/zookeys.1057.65877 (PMC8410751; doi:10.3897/zookeys.1057.65877)

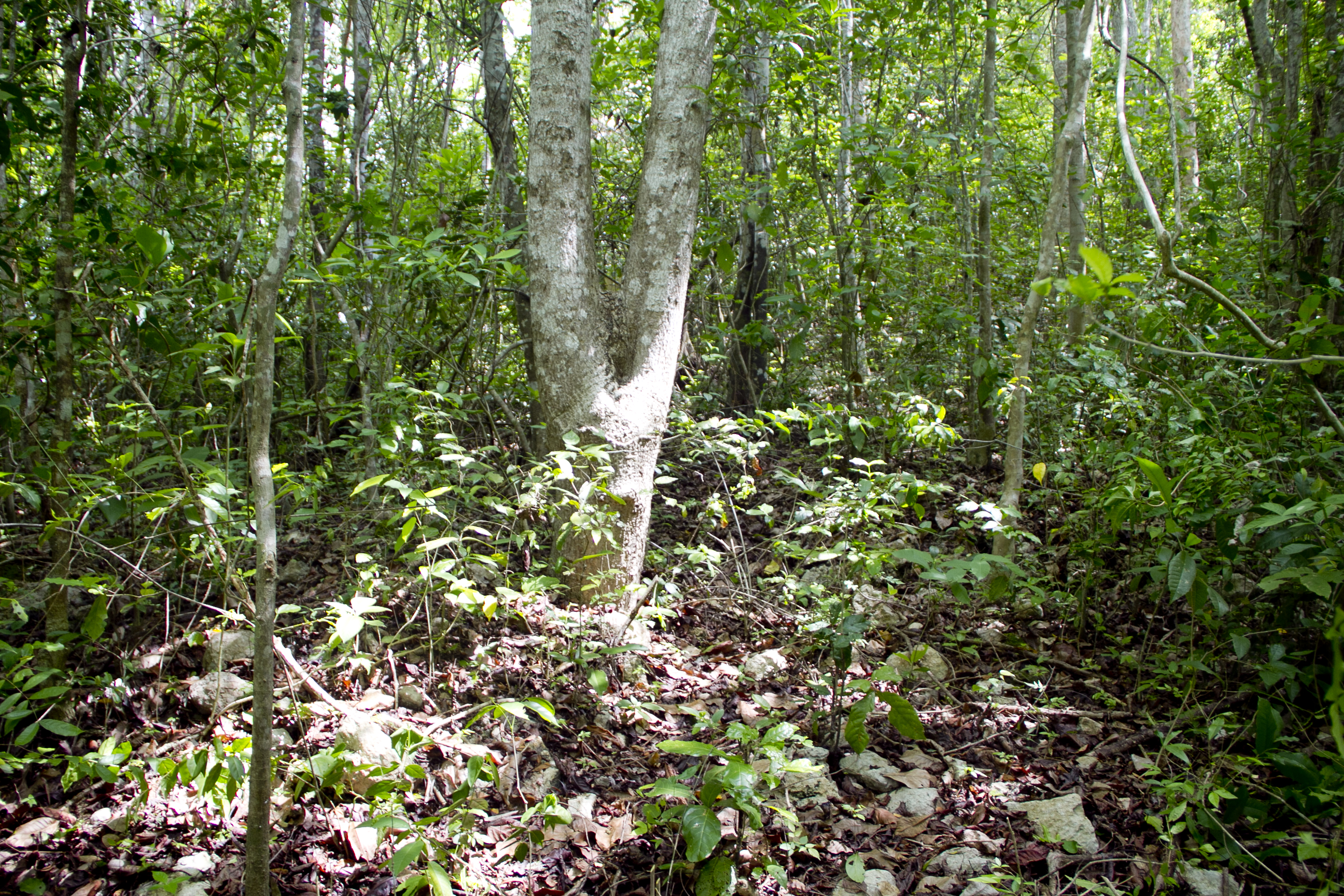

Supplement: Supplementary material 2 — Figure S1 [file zookeys-1057-023-s002.tif]
