# Supplementary material for: Rugitermesursulae (Isoptera, Kalotermitidae), a new drywood termite from the Caribbean coast of Colombia
Source: Zookeys. 2021 Aug 25;1057:23–36. doi: 10.3897/zookeys.1057.65877 (PMC8410751; doi:10.3897/zookeys.1057.65877)
Supplement: Supplementary material 3 — Supplementary references [file zookeys-1057-023-s003.docx]

**Supplementary references**

Abdul R, Parks DH, Willner DL, Engelbrektson AL, Goffredi SK, Warnecke F, Scheffrahn RH, Hugenholtz P (2015) A molecular survey of Australian and North American termite genera indicates that vertical inheritance is the primary force shaping termite gut microbiomes. Microbiome 3: 5. https://doi.org/10.1186/s40168-015-0067-8

Bourguignon T, Lo N, Cameron S, Šobotník J, Hayashi Y, Shigenobu S, Watanabe D, Roisin Y, Miura T, Evans T (2015) The Evolutionary History of Termites as Inferred from 66 Mitochondrial Genomes, Molecular Biology and Evolution 32: 406–421. https://doi.org/10.1093/molbev/msu308

Casalla R, Scheffrahn RH, Korb J (2016b) *Proneotermes macondianus*, a new drywood termite from Colombia and expanded distribution of *Proneotermes* in the Neotropics (Isoptera, Kalotermitidae). ZooKeys 623: 43–60. https://doi:10.3897/zookeys.623.9677

Desai M, Strassert J, Meuser K, Hertel H, Ikeda‐Ohtsubo W, Radek R, Brune A. (2010) Strict cospeciation of devescovinid flagellates and Bacteroidales ectosymbionts in the gut of dry‐wood termites (Kalotermitidae). Environmental Microbiology 12: 2120–2132. https://doi.org/10.1111/j.1462-2920.2009.02080.x

Ghesini S, Simon D, Marini M (2014) *Kalotermes sinaicus* Kemner (Isoptera, Kalotermitidae): new morphological and genetic evidence, and assignment to the new genus *Longicaputermes* gen. nov. Insect. Soc. 61: 123–131. https://doi.org/10.1007/s00040-013-0335-1

Janowiecki M, Scheffrahn R, Austin J, Szalanski A (2020) Population structure of the drywood termite *Incisitermes schwarzi* (Isoptera: Kalotermitidae) in the Carribbean: evidence of arthropogenic dispersal of termites. Journal of agricultural and urban entomology 36: 108. https://doi.org/10.3954/1523-5475-36.1.101

Inward D, Beccaloni G, Eggleton P (2007) Death of an order: a comprehensive molecular phylogenetic study confirms that termites are eusocial cockroaches. Biological Letter 3: 331–335. https://doi.org/doi:10.1098/rsbl.2007.0102

Legendre F, Whiting M, Bordereau C, Cancello E, Evans T, Grandcolas P (2008) The phylogeny of termites (Dictyoptera: Isoptera) based on mitochondrial and nuclear markers: Implications for the evolution of the worker and pseudergate castes, and foraging behaviors, Molecular Phylogenetics and Evolution 48: 615–627. https://doi.org/10.1016/j.ympev.2008.04.017

Maekawa K, Kitade O, Matsumoto T (1999) Molecular Phylogeny of Orthopteroid Insects based on the Mitochondrial Cytochrome Oxidase II Gene. Zoological Science 16: 175–184. https://doi.org/10.2108/zsj.16.175

Monaghan M, Wild R, Elliot M, Fujisawa T, Balke M, Inward, Vogler A (2009) Accelerated Species Inventory on Madagascar Using Coalescent-Based Models of Species Delineation. Systematic Biology 58: 298–311. https://doi.org/10.1093/sysbio/syp027

Ohkuma M, Yuzawa H, Amornsak W, Sornnuwat Y, Takematsu Y, Yamada A, Vongkaluang C, Sarnthoy O, Kirtibutr N, Noparatnaraporn N, Kudo T, Inoue T (2004) Molecular phylogeny of Asian termites (Isoptera) of the families Termitidae and Rhinotermitidae based on mitochondrial COII sequences. Molecular Phylogenetics and Evolution 31: 701–710. https://doi.org/10.1016/j.ympev.2003.09.009

Scheffrahn RH, Bourguignon T, Akama PD, Sillam-Dussès D, Šobotník J (2018) *Roisinitermes ebogoensis* gen. & sp. n., an outstanding drywood termite with snapping soldiers from Cameroon (Isoptera, Kalotermitidae). ZooKeys 787: 91–105. https://doi.org/10.3897/zookeys.787.28195

Thompson G, Miller L, Lenz M, Crozier R (2000) Phylogenetic analysis and trait evolution in Australian lineages of drywood termites (Isoptera, Kalotermitidae). Molecular Phylogenetics and Evolution 17: 419–429. https://doi:10.1006/mpev.2000.0852
